# When Stroke Mimics Peripheral Nerve Injury: A Case Report of Isolated Wrist Drop

**DOI:** 10.31729/jnma.v64i293.9281

**Published:** 2026-01-31

**Authors:** Tai Anjuk Lama, Himal Karki, Kishor Khadka, Rabindara Raj Pandey, Bibek Rajbhandari

**Affiliations:** 1 Department of General Practice and Emergency Medicine, Patan Academy of Health Sciences, Lagankhel, Lalitpur, Nepal; 2 Patan Academy of Health Sciences, Lagankhel, Lalitpur, Nepal; 3 Department of General Practice and Emergency Medicine, Tribhuvan University Teaching Hospital, Maharajgunj, Kathmandu, Nepal

**Keywords:** *peripheral nerve injury*, *stroke*, *wrist-drop*

## Abstract

Isolated wrist drop as a presentation of stroke is uncommon and may pose a diagnostic dilemma. We present a case of a 65-year-old man with hypertension and diabetes who presented with sudden-onset weakness of the left hand and inability to extend fingers, without any other focal neurological deficits. Noncontrast computed tomography of the head was normal. However, diffusion-weighted magnetic resonance imaging of the brain revealed an acute ischemic infarct involving the right frontoparietal and occipital lobes. A diagnosis of isolated left-sided wrist drop secondary to acute ischemic stroke was made. The patient showed clinical improvement with antiplatelet and statin therapy, along with physiotherapy, on follow-up. Although rare, isolated wrist drop may be a manifestation of stroke, and clinicians must remain vigilant to avoid diagnostic delays and to administer appropriate treatment in a timely manner.

## INTRODUCTION

Stroke, a leading global cause of morbidity and mortality, affects approximately 12.2 million individuals annually, with an estimated 101 million people living with it.^1^ While stroke commonly presents with a range of neurological deficits, isolated wrist drop is rare (approximately 1%), often attributed to radial nerve injury.^2^ However, strokes involving parietal lobe/central sulcus may selectively impair wrist extensor function without sensory loss.^3^ Prompt differentiation is crucial, as central etiologies require thrombolysis within 4.5 hours of symptom onset.^4^ We report a case of a 65-year-old man with sudden-onset left-sided wrist drop who was subsequently diagnosed with acute ischemic stroke on neuroimaging. This case highlights the importance of considering stroke in the differential diagnosis of sudden-onset isolated wrist drop to facilitate timely neuroimaging, accurate diagnosis and appropriate management.

## CASE REPORT

**Figure 1:**
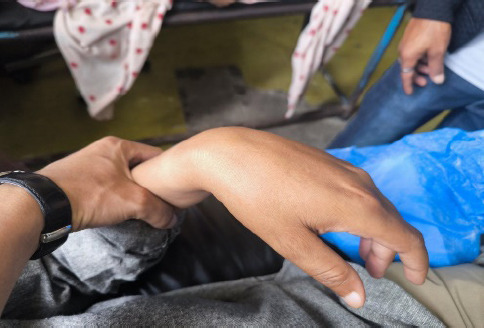
Isolated left wrist drop.

A 65-year-old man with a history of hypertension and diabetes, and a current smoker, presented to the emergency department with sudden-onset left-hand weakness and inability to extend the left wrist and fingers for six hours (Figure 1). There was no history of trauma or trivial falls, loss of consciousness, slurring of speech, facial deviation, abnormal gait, abnormal body movements, dizziness, visual disturbances, fever, nausea, vomiting, or presyncopal symptoms. On examination, the patient was alert with a Glasgow Coma Scale (GCS) score of 15/15, and bilateral pupils were round, regular and reactive to light. He was afebrile, with a blood pressure of 110/80 mmHg and a pulse rate of 100 beats per minute. Motor examination revealed normal muscle power (5/5 on the Medical Research Council [MRC] scale) in all extremities except at the left wrist and left-hand fingers, where extension was absent (0/5) while flexion was preserved (5/5). Sensory examination was intact and bilateral plantar reflexes were downgoing. No other focal neurological deficits were present. Other relevant systemic examinations were within normal limits.

**Figure 2:**
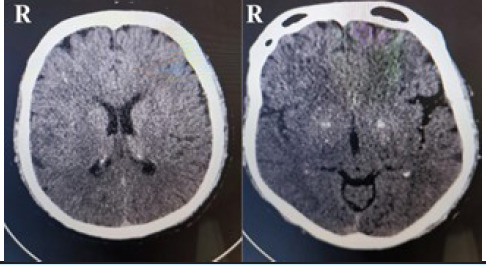
Non-contrast enhanced computed tomography (NCCT) showed no acute abnormalities.

**Figure 3:**
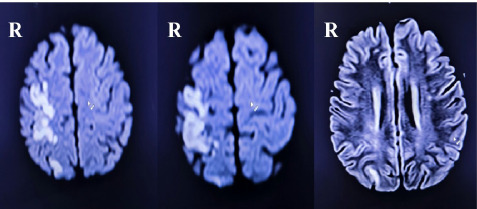
Magnetic resonance imaging (MRI) of the brain in diffusion-weighted imaging (DWI) sequence showing acute ischemic infarct involving right frontoparietal and occipital lobes.

An X-ray of the left wrist and hand revealed no gross abnormalities. Routine laboratory investigations were within normal limits. Electrocardiogram showed normal sinus rhythm. Given the diagnostic dilemma relating to underlying cause, an immediate non-contrast enhanced computed tomography (NCCT) scan of head was performed, which showed no acute abnormalities (Figure 2). Subsequent diffusion-weighted imaging (DWI) sequence of magnetic resonance imaging (MRI) of the brain revealed an acute ischemic infarct involving right frontoparietal and occipital lobes, suggesting involvement of multiple small-vessel vascular territories (Figure 3). Based on clinical and radiological findings, a diagnosis of central isolated wrist drop secondary to ischemic stroke was made.

The patient was given a loading dose of aspirin 300mg and atorvastatin 80mg, along with application of wrist splint. Thrombolytic therapy was not administered due to delayed presentation beyond therapeutic window (after 4.5 hours of onset of symptoms). A neurosurgical consultation was obtained and mechanical thrombectomy was discussed but not pursued. After 24 hours of observation, the patient was discharged with advice to continue his regular antihypertensive and antidiabetic medications and aspirin 75mg and atorvastatin 40mg once daily, and to continue hand-strengthening exercises.

Carotid Doppler ultrasonography done on day 3 of follow-up revealed a homogenous atherosclerotic plaque in the right proximal internal carotid artery (ICA) causing 22.5% luminal narrowing, and a calcified plaque (measuring 2mm × 3.9mm) in the right distal common carotid artery (CCA). An echocardiogram revealed mild concentric left ventricular hypertrophy (LVH) with preserved systolic function, no regional wall motion abnormality, and no evidence of intracardiac thrombus, mass or valvular vegetation. At subsequent follow-up visits at 2 weeks and 4 weeks, the patient showed continued clinical improvement with progressive recovery of the wrist and finger extension strength improving to 3/5 at 2 weeks and further to 4/5 at 4 weeks.

## DISCUSSION

Isolated distal limb weakness is most commonly caused by peripheral etiologies involving nerves or muscles and is only rarely due to central causes such as stroke. Distal monoparesis confined to the hand or wrist has been reported in only a small proportion of ischemic strokes and is therefore frequently misdiagnosed as peripheral nerve injury.^2,4^ Furthermore, a study by Harada et al. concluded that atypical presentations in medical practice are highly likely to be associated with diagnostic errors.^5^ In our patient, isolated wrist drop was initially misleading and was later found to be caused by ischemic stroke on neuroimaging. Consequently, rare and atypical presentations of stroke may lead to misdiagnosis and delays in appropriate management.

Because of the atypical presentation, the cause of wrist drop in our patient was initially attributed to a peripheral etiology, most likely radial nerve palsy. Peripheral nerve injuries typically result in simultaneous motor and sensory deficits, and radial nerve injury classically presents with loss of wrist and finger extension accompanied by sensory loss over the dorsum of the hand.^6^ In contrast, our patient presented with isolated motor weakness with preserved sensation and normal X-ray findings of the hand. The abrupt onset of symptoms in the presence of hypertension and diabetes further raised suspicion of a central etiology. As highlighted by Edlow et al., sudden-onset focal neurological deficits should prompt consideration of stroke, particularly in patients with underlying vascular risk factors.^7^ The presence of these clinical features in our patient prompted further evaluation with neuroimaging.

Neuroimaging was crucial in establishing the diagnosis in our patient. While the NCCT scan of the head revealed no acute abnormalities and excluded intracranial hemorrhage (ICH), diffusion-weighted sequence of MRI of the brain revealed cortical infarction involving the right frontoparietal and occipital lobes, consistent with involvement of multiple small-vessel vascular territories. DWI MRI is highly sensitive for the detection of acute ischemic stroke and can identify infarction within minutes of symptom onset, often when CT imaging remains normal.^8,9^ Cortical involvement of the right frontoparietal region, likely affecting the lateral aspect of the precentral gyrus corresponding to the hand motor representation, provides a clear anatomical basis for the patient’s contralateral isolated wrist drop with preserved sensation, as lesions in this area can selectively impair wrist and finger extensor function without sensory involvement.^2-4^ This case underscores that isolated wrist drop can occur despite radiological evidence of multiterritory cortical infarction.

Multiterritory cortical infarctions are commonly associated with cardioembolic events or artery-to-artery embolism, particularly in patients with vascular risk factors such as hypertension, diabetes and smoking.^10,11^ In our patient, echocardiography demonstrated mild concentric LVH with preserved systolic function, no regional wall motion abnormality, and no evidence of intracardiac thrombus, mass or valvular vegetation, making a cardioembolic source unlikely. Carotid Doppler ultrasonography revealed atherosclerotic plaques with luminal narrowing in the right proximal ICA and right distal CCA. Although these findings suggest a possible artery-to-artery embolic mechanism, a definite etiological source could not be identified.

Despite radiological involvement of the occipital lobe, our patient did not demonstrate corresponding visual symptoms suggesting the presence of a clinically silent infarct. Silent infarcts have been reported in approximately 10% to 20% of patients with stroke and are often detected incidentally on neuroimaging.^12^ Although asymptomatic, these infarcts are clinically relevant, as they are associated with an increased risk of future symptomatic strokes and cognitive impairment.^13^

After ICH was excluded, the patient was treated with a loading dose of antiplatelet therapy and a high-intensity statin as per hospital stroke management protocol. These medications were continued after discharge as secondary prevention measures. Evidence from a recent Cochrane review indicates that early antiplatelet therapy in acute ischemic stroke reduces mortality and dependency with reduction in the risk of recurrent ischemic attacks improving long-term outcomes.^14^ In our patient, thrombolytic therapy was not administered due to the delayed presentation and mechanical thrombectomy was not pursued because the infarction involved multiple small-vessel vascular territories rather than a single large vessel.

Accurate diagnosis of isolated wrist drop secondary to stroke requires a high level of clinical expertise and a high degree of clinical suspicion, often supported by advanced neuroimaging. As the management of peripheral and central causes of wrist drop differs significantly, accurate localization of the pathology is essential for initiation of appropriate treatment and secondary prevention.

## CONCLUSION

Isolated wrist drop is a rare but clinically important manifestation of ischemic stroke that can be mistaken for peripheral nerve injury. Sudden-onset focal motor deficits, particularly in patients with cardiovascular risk factors should prompt consideration of a central etiology even in the absence of other neurological signs. Stroke should be considered until proven otherwise and diagnosis requires advanced neuroimaging such as diffusion-weighted MRI. Heightened clinical awareness of this rare presentation is essential to avoid delayed diagnosis, timely management and effective secondary prevention.
